# Identification and Verification of Immune Metabolism–Related Biomarkers and Immune Infiltration Landscape for Pediatric Opsoclonus Myoclonus Ataxia Syndrome in Neuroblastoma

**DOI:** 10.1111/cns.70610

**Published:** 2025-11-04

**Authors:** Minglei Li, Jinlei Li

**Affiliations:** ^1^ Department of pediatric internal Medicine one Weifang People's Hospital Weifang Shandong China; ^2^ Department of Neurosurgery Three Weifang People's Hospital Weifang Shandong China

**Keywords:** diagnosis, immune infiltration, machine learning, opsoclonus myoclonus ataxia syndrome

## Abstract

**Purpose:**

This study aims to screen immune metabolism‐associated biomarkers for pediatric opsoclonus myoclonus ataxia syndrome (OMAS) in neuroblastoma.

**Methods:**

Immune metabolism–related genes were retrieved from the GeneCards database. The differentially expressed immune metabolism–related genes in OMAS were identified by bioinformatics, immune infiltration, and WGCNA analyses. The diagnostic genes were screened by three machine learning algorithms and validated by ROC curve and nomogram model. Correlation between diagnostic genes and differential immune infiltrated cells, GSEA, and drug chemistry small‐molecule analyses was performed. Lastly, validation was performed in eight paired clinical samples.

**Results:**

Total 162 differentially immune metabolism–related genes were obtained. Four diagnostic genes were selected by machine learning methods. The predictive accuracy of biomarker genes for OMAS was determined by nomograms and calibration curves. The targeted drugs for the four diagnostic genes contained bardoxolone methyl, alogliptin, and teneligliptin. Finally, clinical validation showed TRAF3IP2, DPP4, and RIPK1 upregulation and KEAP1 downregulation, consistent with bioinformatics analysis. The predictive accuracy of biomarkers was validated by ROC curve in clinical samples.

**Conclusion:**

Four immune metabolism–associated diagnostic genes were identified, including TRAF3IP2, RIPK1, KEAP1, and DPP4 for OMAS.

## Graphical abstract

Based on the GEO database and GeneCards database, 1785 immune metabolism genes, 1879 DEGs, and 1560 module genes were screened out. After taking the intersection, 162 differential immune metabolic–related genes were obtained. Four diagnostic biomarker genes, TRAF3IP2, RIPK1, KEAP1, and DPP4, were screened out through three machine learning algorithms and verified by ROC curve and nomogram model. Finally, clinical validation demonstrated that compared with the control group, DPP4, RIPK1, and TRAF3IP2 in the OMAS group were significantly upregulated at the mRNA and protein levels, while KEAP1 was significantly downregulated, which was consistent with the bioinformatics analysis. The ROC curve in clinical samples verified the predictive accuracy of the biomarkers.

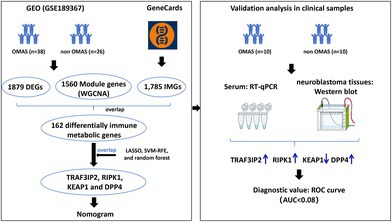

## 1 Introduction

Opsoclonus‐myoclonus‐ataxia syndrome (OMAS) is an uncommon neurological disorder, and it is most common in young children [1]. This situation is usually manifested with a combination of characteristic eye movement disorders and myoclonus, as well as ataxia, irritability, and sleep disorders, with a propensity for relapse [2]. OMAS in children mostly occurs between 18 and 24 months old [3]. Most of the pediatric OMAS is associated with neuroblastoma [4, 5]. OMAS with neuroblastoma usually has a good prognosis and a higher survival rate after treatment than even neuroblastoma without OMAS [6]. OMAS often leads to ‌precipitous‌ diagnosis of underlying tumors. Thus, it is imperative to diagnose OMAS early in neuroblastoma for effective prevention.

Recent studies have revealed that the metabolic alterations of immune cells play a determinant role in hastening the development of neurological diseases, including OMAS [7, 8]. For instance, Pranzatelli et al. [8] found that even though the majority of children with OMAS displayed typical levels of cerebrospinal fluid (CSF) cells, they showed elevated numbers of CD19 B cells (up to 29%) and gamma delta T cells (up to 26%), along with reduced percentages of CD4 T cells and the CD4/CD8 ratio, which persisted for many years following the onset of the condition and standard therapies. A previous study uncovered that the CSF macrophage marker sCD14 concentration was 1.9‐fold higher in other inflammatory neurological disorders and 1.4‐fold higher in OMAS than in controls [9]. These results emphasize the significant role of the immune system in OMAS. Hence, conducting thorough investigations into characteristic genes associated with the immune microenvironment is crucial for identifying OMAS patients who could potentially benefit from immunotherapy. However, the diagnostic performance of immune metabolic–related biomarkers for OMAS has not been clarified.

**FIGURE 1:**
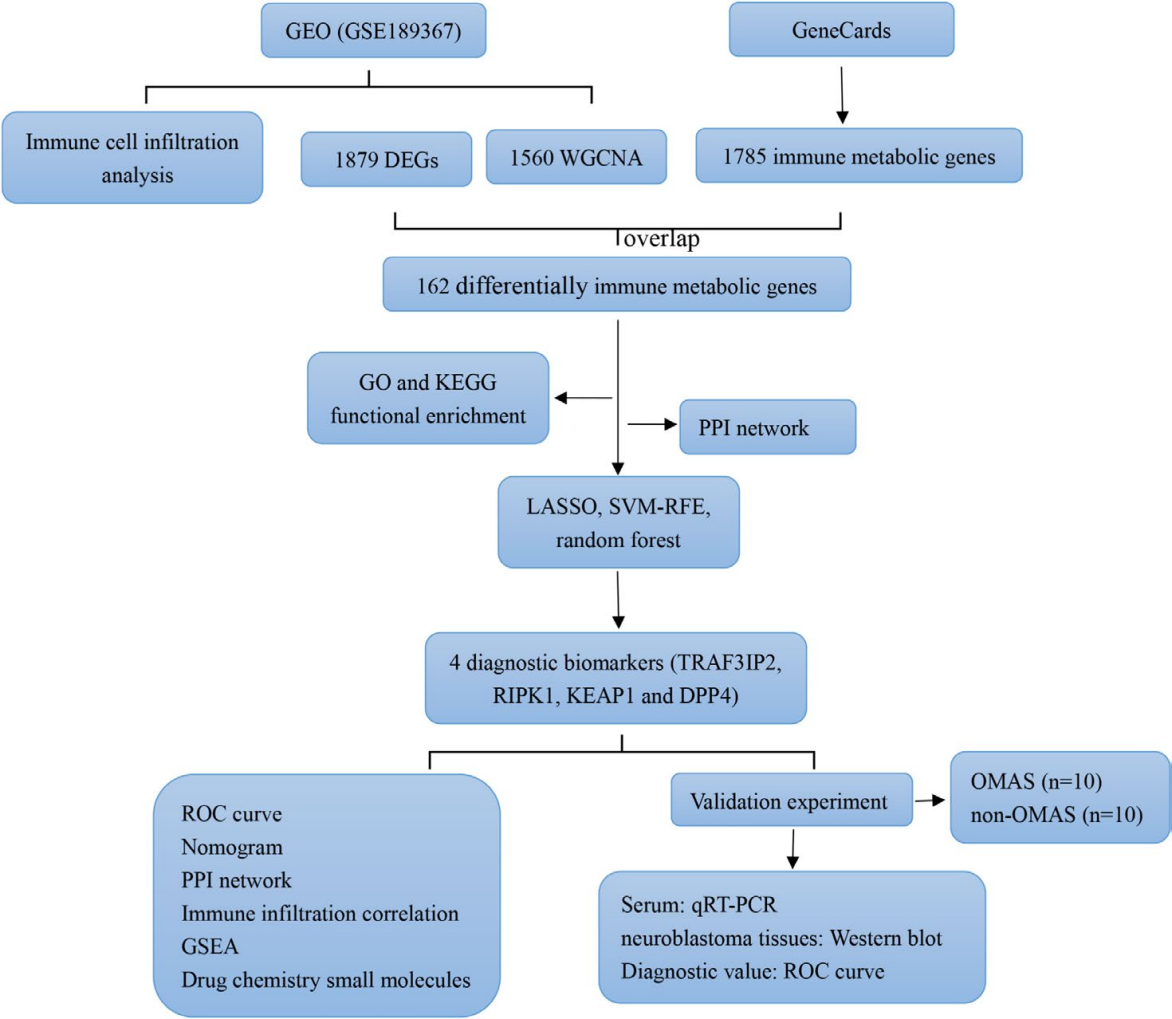
The workflow diagram of this research.

Therefore, in this study, we aimed to screen immune metabolism‐associated diagnostic biomarkers for OMAS in children by implementing machine learning methods, and predict small potential drug molecules by targeting biomarkers for OMAS in children. It is anticipated that our study will lay the foundation for the diagnosis and treatment of OMAS in children. The workflow of this study is shown in Figure 1.

## 2 Materials and Methods

### 2.1 Data Acquisition and Preprocessing

GSE189367 dataset was obtained from the GEO database [10], including 38 neuroblastoma samples from patients with OMAS and 26 neuroblastomas from patients without OMAS (controls). The original quantitative count values of genes for each sample were downloaded, and the “TMM” algorithm in the “edgeR” package [11] was employed to standardize the raw count, followed by converting it into logCPM values for subsequent analysis.

### 2.2 Immune Cell Infiltration Analysis

The 28 types of immune cells and corresponding marker genes were acquired from previous literature, and the gene expression matrix of 26 controls and 38 OMAS samples was acquired. “GSVA” package [12] was employed to calculate the fraction of infiltrated immune cells, and the Wilcoxon test was utilized to compare the differences in fractions of immune cells between control and OMAS samples.

### 2.3 Identification of Differentially Expressed Genes (DEGs)

DEGs between OMAS samples and controls were analyzed by utilizing the “edgeR” package. DEGs meeting the criteria of *p* < 0.05 and |log2FC| > 0.585 were identified.

### 2.4 Weighted Gene Co‐Expression Network Analysis (WGCNA)

Based on the expression values of all genes in each sample, the enrichment scores of differential immune cells in each sample were extracted. The R package “WGCNA” [13] was utilized to conduct modular clustering analysis on genes to identify gene set modules with high synergistic changes. First, to achieve a scale‐free network distribution as much as possible, the “power” value was utilized to compute the square of the correlation coefficient between connectivity (*k*), p (*k*), and average connectivity. Using clustering and dynamic pruning techniques, parameters such as minModuleSize = 30 (ensuring that each module consists of at least 30 genes) and MEDisThres = 0.3 (merging modules with similarity above 0.7) were established to group genes with high correlations into modules. Lastly, modules strongly linked to various immune cells (with a correlation coefficient *r* > 0.3 and *p* < 0.05) were chosen as those specifically related to immunity.

### 2.5 Acquisition of Differentially Expressed Immune Metabolism Genes

First, immune metabolism genes were retrieved from “GeneCards” database [14] with the score > 8. Subsequently, intersection analysis was performed on immune metabolism genes with DEGs and the significant module genes related to differential immune infiltrated cells, followed by GO and KEGG pathway enrichment analysis.

### 2.6 Identification of Diagnostic Biomarkers via Machine Learning Methods

Based on the expression values of differentially immune metabolism genes obtained above, three machine learning algorithms were utilized to screen key genes, including LASSO logistic regression model, SVM‐RFE model and random forest model. (a) LASSO logistic regression model: the “glmnet” package [15] in R was utilized to identify the feature genes, and the parameters were conducted as family = “binomial”, type. measure = “class”, nfold = 10. (b) SVM‐RFE model: the differentially immune‐related immune metabolism genes were sorted using “SVM” algorithm [16] in R package “e1071”, and Recursive Feature Elimination (RFE) method was employed to acquire the importance and ranking of each gene, and the error rate and accuracy of each iteration combination was obtained. The lowest error rate as the best combination was selected, and the selected genes were screened as the feature genes. (c) Random forest model: random forest method in R package “randomForest” [17] was utilized to obtain feature genes based on the differentially expressed immune metabolism genes. Then the “Mean DecreaseAccuracy” and “Mean Decrease Gini” methods were employed to sort the genes obtained from the “RF” algorithm according to their importance level, and the TOP20 genes were selected as feature genes. In addition, the common genes obtained from three machine learning algorithms were acquired as the biomarkers, then the multiple logistic regression was applied to calculate the regression coefficients of each diagnostic gene, and the risk scores were calculated following the formula: Risk score = ∑*β*_gene_ × Exp_gene_ (where *β*_gene_ indicates the regression coefficient, Exp_gene_ indicates the expression levels of genes in various samples). Besides, the ROC curve was drawn to assess the diagnostic value of biomarker genes. Then, the Spearman correlation analysis was performed to evaluate the association between biomarker genes and differential immune cells.

### 2.7 Nomogram Construction

The “rms” package [18] was utilized to build a nomogram, then the calibration and DCA curves were drawn to evaluate the predictive ability of thenomogram.

### 2.8 Protein–Protein Interaction (PPI) Network

The “STRING” database [19] was used to search the protein interaction pairs of the diagnostic genes with the cutoff value of PPI score ≥ 0.4. Subsequently, the “GeneMANIA” database [20] was applied to build the PPI network of diagnostic genes.

### 2.9 Gene Set Enrichment Analysis (GSEA)

The Pearson correlation coefficients between each diagnostic gene and all other genes in OMAS samples were calculated. Then “clusterProfiler” was employed to conduct the GSEA enrichment analysis with the cutoff values of *p*.adjust < 0.05. The Benjamini‐Hochberg method was utilized for multiple test correction, and the corrected *p* value was obtained.

### 2.10 Drug Chemistry Small Molecules

“DGIdb” database is a collection of drug–gene interactions [21, 22] and was applied to predict the targeted drugs for the diagnostic genes. The drug–gene network was constructed by using “Cytoscape” software [23].

### 2.11 Clinical Samples

Ten paired samples (a total of 20 cases) were collected from each of the children with neuroblastoma diagnosed with OMAS and non‐OMAS. For each case, serum (for detecting the mRNA expression levels of TRAF3IP2, RIPK1, KEAP1, and DPP4) and surgically resected neuroblastoma tissues (for detecting the expression of corresponding proteins) were collected. Basic information of patients was collected, including age, gender, history of hepatitis A and meningococcal vaccination, and tumor location. All human studies have been reviewed by the ethics committees of Weifang People's Hospital (KYLL20220622‐2) and have all met the ethical standards stipulated in the Helsinki Declaration (Brazilian version, 2013, revised edition). Informed consent was obtained from all individual participants included in the study.

### 2.12 Real Time Quantitative PCR Assay (RT‐qPCR)

To verify the differential expression of diagnostic biomarkers (DPP4, KEAP1, RIPK1, and TRAF3IP2) between OMAS samples and controls, RT‐qPCR was performed. Briefly, RNA samples were isolated using Trizol reagent. The first‐strand cDNA was synthesized using the cDNA Synthesis Kit (Invitrogen). The primer sequences are shown in Table 1.

**TABLE 1:** The primer sequences.

Gene	Sequences (5′‐3′)
DPP4 (F)	ATTCCGTACCCAAAGGCAGG
DPP4 (R)	AGGCCACGTCACACAAGTAG
KEAP1 (F)	ACGGGACAAACCGCCTTAAT
KEAP1 (R)	ATACAGTTGTGCAGGACGCA
RIPK1 (F)	TATCCCAGTGCCTGAGACCAAC
RIPK1 (R)	GTAGGCTCCAATCTGAATGCCAG
TRAF3IP2 (F)	CTGCGTCTGAGTCTGTGGTT
TRAF3IP2 (R)	TATCCCGTGTCTATGGTTGG

### 2.13 Western Blot Assay

Neuroblastoma tissues were homogenized, followed by protein extraction using a protein extraction kit (Beyotime, Shanghai, China). After quantification, total proteins were separated by the SDS‐PAGE system, and the identified protein bands were transferred onto PVDF membranes. Nonspecific binding sites were blocked using skim milk. Subsequently, immune reactions were conducted with primary antibodies against DPP4, KEAP1, RIPK1, and TRAF3IP2 (1:1000) and the secondary IgG antibodies (1:5000). All the antibodies were purchased from ThermoFisher Scientific, USA. Protein bands were stained by enhanced chemiluminescence reagent. ImageJ software was used for quantitative analysis.

### 2.14 Statistical Analysis

The continuous data were expressed as mean ± standard deviation, and the categorical data were displayed as numbers. Statistical analysis was performed by SPSS 18.0 software and GraphPad Prism. All continuous variables were evaluated for normality through the Shapiro–Wilk test. For the data conforming to the normal distribution, the Student *t*‐test was used for comparison between the two groups. Data with non‐normal distribution were tested using the Mann–Whitney U test. Categorical variables were tested by Chi‐square test or Fisher's exact test. Differences with *p* < 0.05 was considered significant.

## 3 Results

### 3.1 Immune Cell Infiltration and DEGs

**FIGURE 2:**
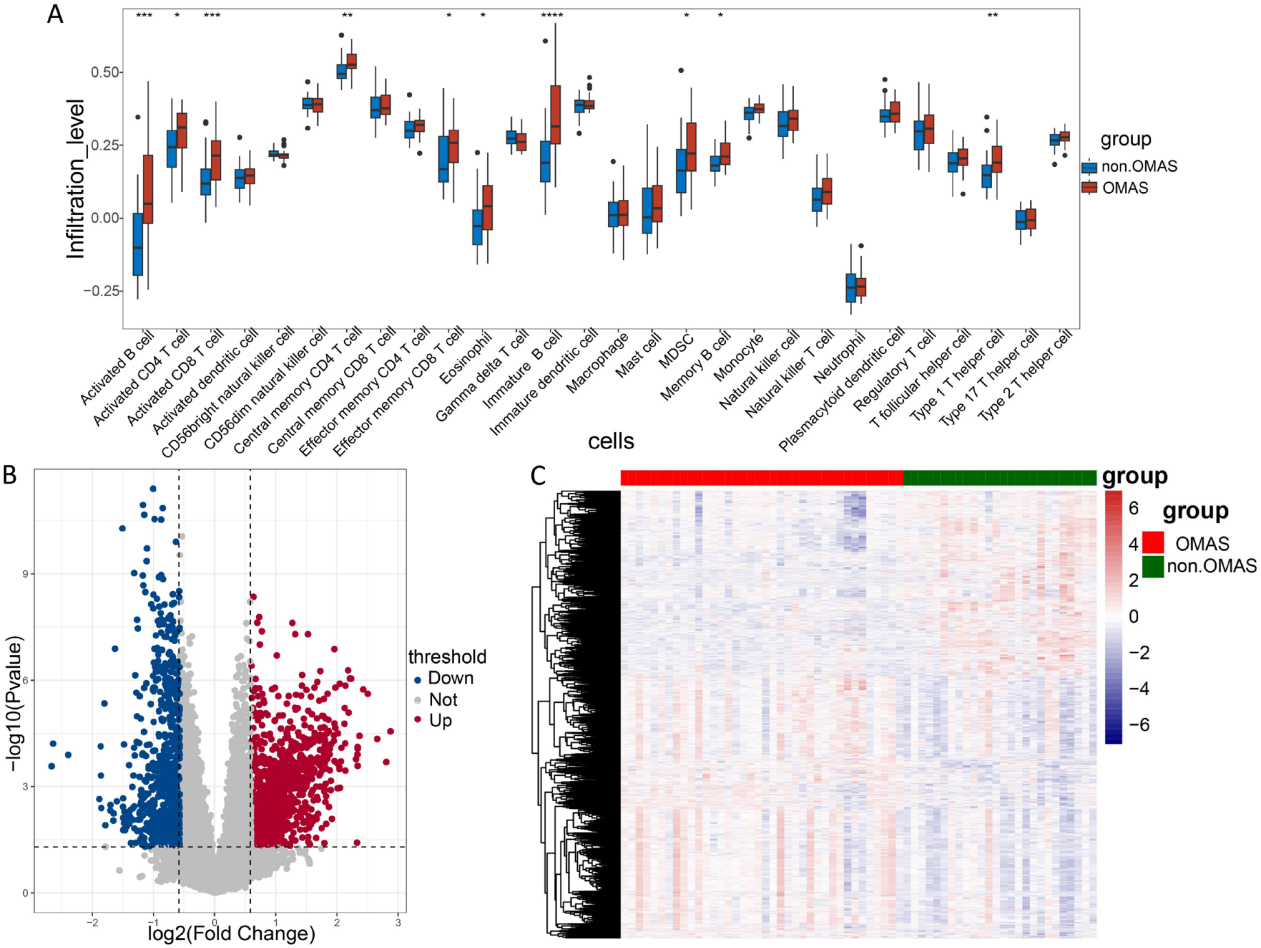
Immune cell infiltration and differentially expressed genes (DEGs). (A) Differences in the fraction of immune cells between control and opsoclonus myoclonus ataxia syndrome (OMAS) groups. Volcano plot (B) and heatmap (C) of DEGs obtained between control and OMAS groups. *p < 0.05, **p < 0.01, ***p < 0.001, and ****p < 0.0001.

Figure 2A depicts that a total of 10 types of immune cells exhibited differential infiltration between OMAS and control samples (*p* < 0.05), such as macrophages, immature B cells, and eosinophils. Additionally, a total of 1879 DEGs were screened, containing 1104 upregulated and 775 downregulated ones (Figure 2B,C).

### 3.2 Identification of Differential Immune Infiltrated Cells Related Genes

**FIGURE 3:**
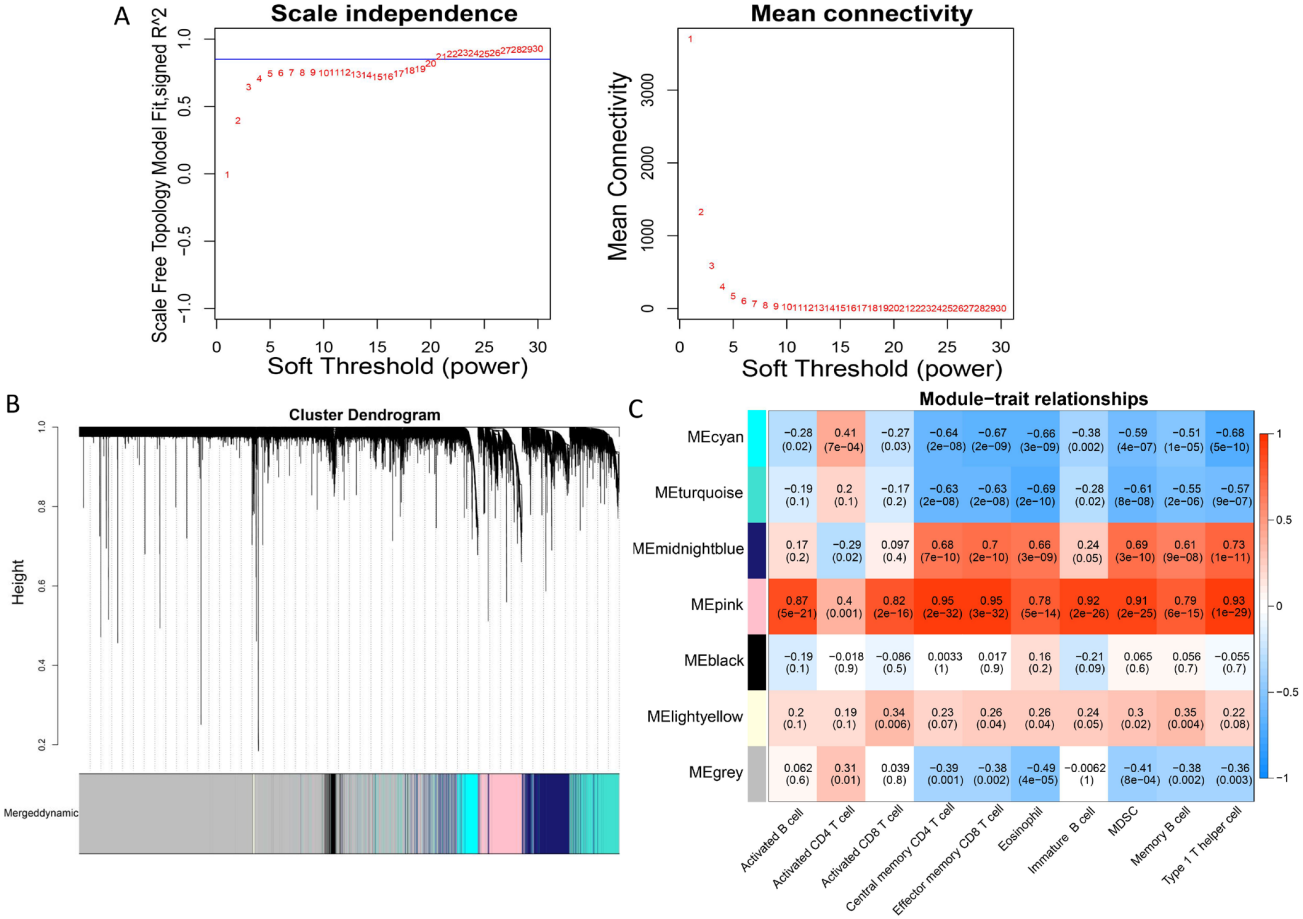
Weighted gene co‐expression network analysis. (A) Hierarchical clustering of samples and selection of the weight parameter “power” of adjacency matrix and the mean connectivity. (B) Tree diagram for module division. (C) Global outline of the relationship between the modules and immune cells.

To maximize the fulfillment of the scale‐free network distribution premise, the “power” of 21 was selected as the optimal soft‐threshold (Figure 3A). A total of six modules were integrated (Figure 3B). In addition, the absolute correlation coefficients between the pink module (1560 genes) and 10 differentially expressed immune cells were all above 0.3; therefore, the pink module genes were considered differential immune infiltration–related genes (Figure 3C).

### 3.3 Acquisition of Differentially Immune Metabolism–Related Genes and PPI Network

**FIGURE 4:**
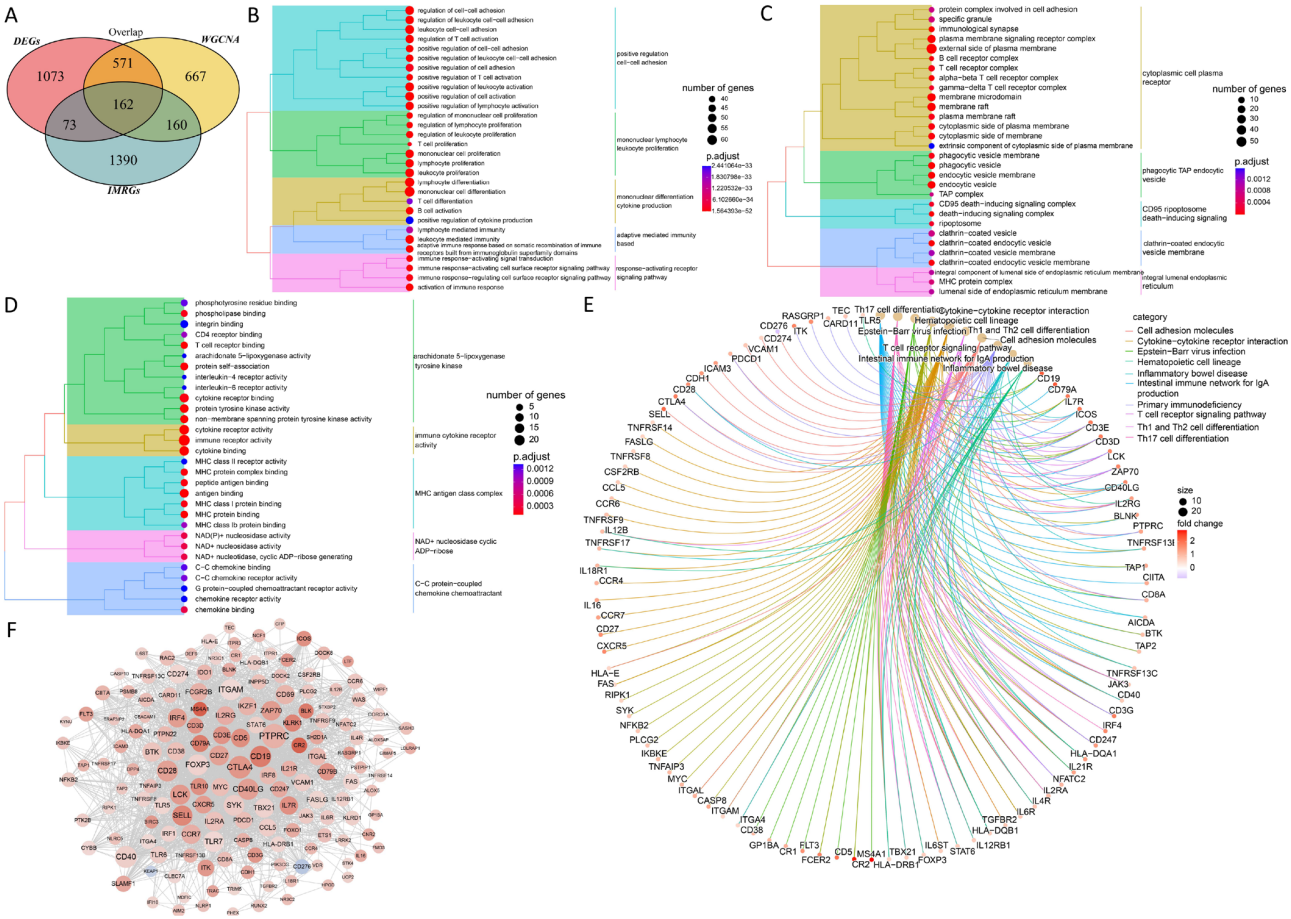
Acquisition of differentially immune‐related immune metabolism genes and PPI network. (A) Differentially immune‐related immune metabolism genes. GO functional enrichment analysis of differentially immune‐related immune metabolism genes, GO‐BPs (B), GO‐CCs (C), GO‐MFs (D). (E) Kyoto encyclopedia of genes and genomes (KEGG) enrichment analysis. (F) PPI network.

A total of 1785 immune metabolism genes were searched in “GeneCards” database. By intersection analysis of DEGs, immune metabolic genes, and module genes, 162 overlapped genes were obtained as the differentially immune metabolic genes (Figure 4A). A list of 162 differential immune metabolic genes is shown in Appendix S1. Functional enrichment analysis showed that the 162 overlapping genes were involved in 1252 GO‐BPs, 60 GO‐CCs, 102 GO‐MFs, and 61 KEGG pathways, such as positive regulation of cell–cell adhesion (BP) (Figure 4B), cytoplasmic cell plasma (CC) (Figure 4C), immune cytokine receptor activity (MF) (Figure 4D) and cytokine‐cytokine receptor interaction–related pathway (Figure 4E). Besides, the PPI network of the differentially immune metabolism genes was constructed, which was comprised of 2246 PPI interactions and 157 nodes (Figure 4F).

### 3.4 Diagnostic Genes Screening and Diagnostic Model Construction

**FIGURE 5:**
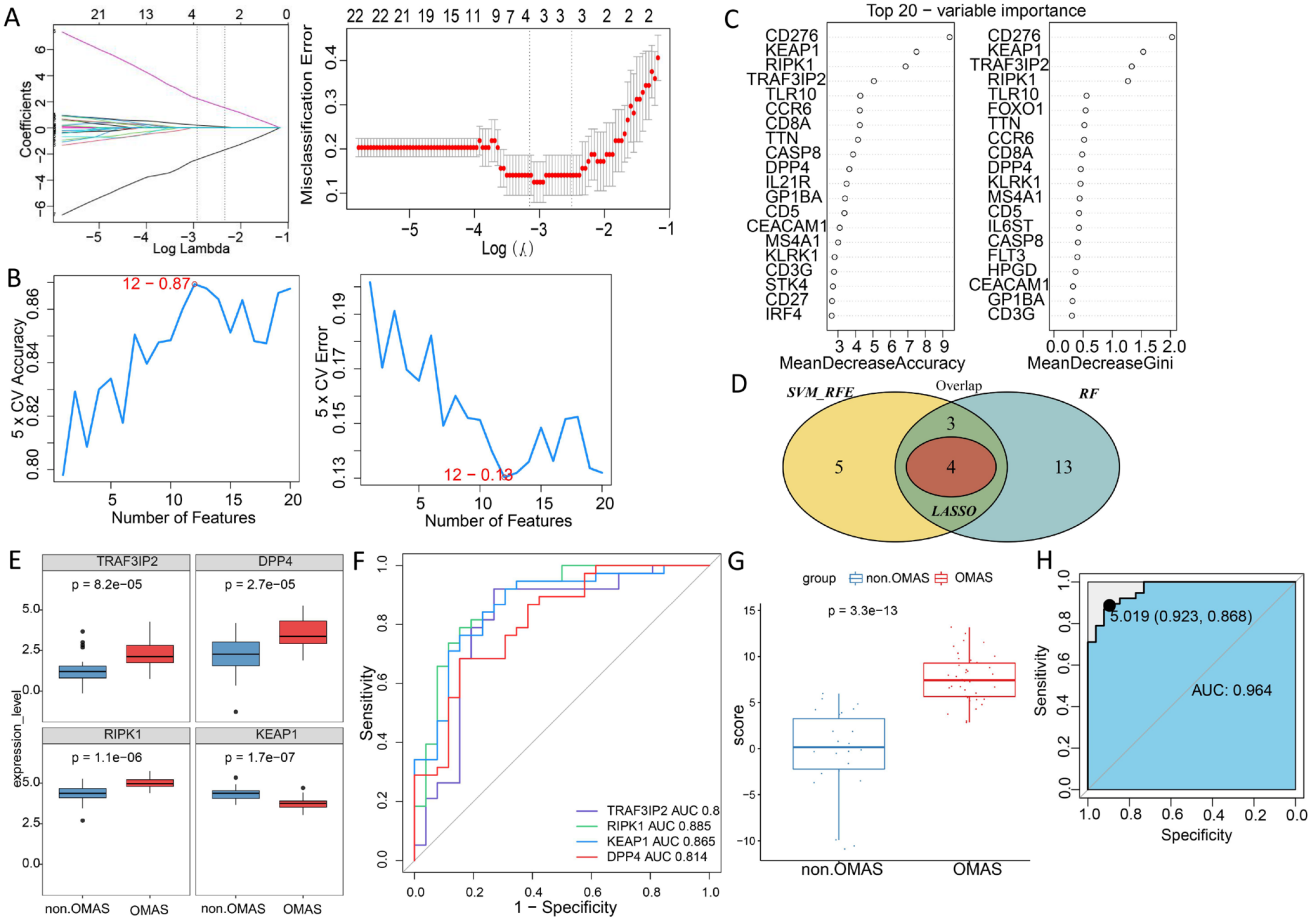
Identification of diagnostic genes. Feature genes were obtained using the LASSO logistic regression model (A), SVM‐RFE model (B), and random forest model (C). (D) Venn diagram. (E) The expression level of four diagnostic genes in control and OMAS groups. (F) Receiver operating characteristic (ROC) curve of 4 diagnostic genes. (G) Diagnostic score in control and OMAS groups. (H) ROC curve of diagnostic score.

Based on the expression values of differentially immune metabolism genes obtained above in various samples, the feature genes were obtained using the LASSO logistic regression model (*n* = 4, Figure 5A), SVM‐RFE model (*n* = 12, Figure 5B), and random forest model (*n* = 20, Figure 5C). Moreover, 4 common genes obtained from three machine learning algorithms were acquired as the diagnostic biomarkers (Figure 5D), including TRAF3IP2, RIPK1, KEAP1, and DPP4. The diagnostic score was established utilizing the formula: Risk Score = (−0.0236) * TRAF3IP2 + 5.233 * RIPK1 + (−5.676) * KEAP1 + 0.769 * DPP4. Additionally, the ROC curve was drawn to assess the diagnostic value of biomarkers and risk score; the results showed that AUCs of diagnostic gene expression and risk score were all above 0.8 (Figure 5E–H), which indicated that the four diagnostic biomarkers had good diagnostic performance.

### 3.5 Nomogram

**FIGURE 6:**
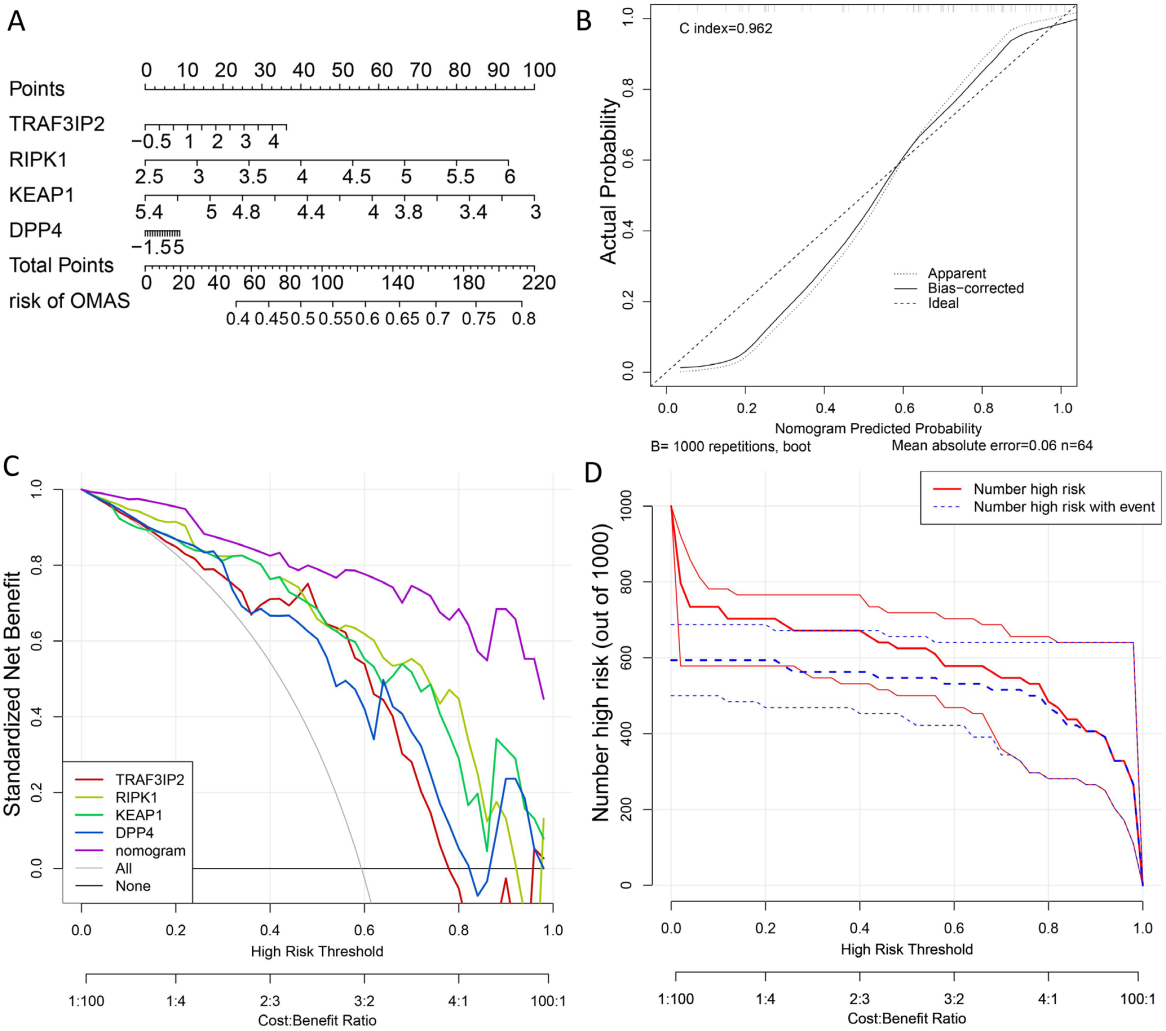
Construction of a nomogram (A) Opsoclonus Myoclonus Ataxia Syndrome diagnostic nomogram. (B) Calibration curve. (C) Decision curve analysis (DCA) curve evaluated the clinical value of the nomogram. (D) Evaluation of the clinical impact curve of the nomogram based on the DCA curve.

A nomogram of diagnostic genes was constructed (Figure 6A) and a calibration curve was plotted to assess the predictive capability of the nomogram (Figure 6B). The findings showed that the C‐index equaled 0.962, implying that the nomogram possessed high accuracy for predicting OMAS. Moreover, the DCA curve demonstrated that the nomogram curve was above the gray line curve, denoting that the clinical benefits of the patients' nomogram were superior (Figure 6C). To evaluate the clinical effectiveness of the nomogram more intuitively, a clinical impact curve was generated based on the DCA curve. The “Number high risk” curve closely approximated the “Number high risk with event” curve when the high‐risk threshold ranged of 0.8–1, signifying that the nomogram possessed promising predictive ability (Figure 6D).

### 3.6 PPI Network, Correlation Between Four Diagnostic Genes and Differential Immune Cells, GSEA, and Drug Chemistry Small Molecules

**FIGURE 7:**
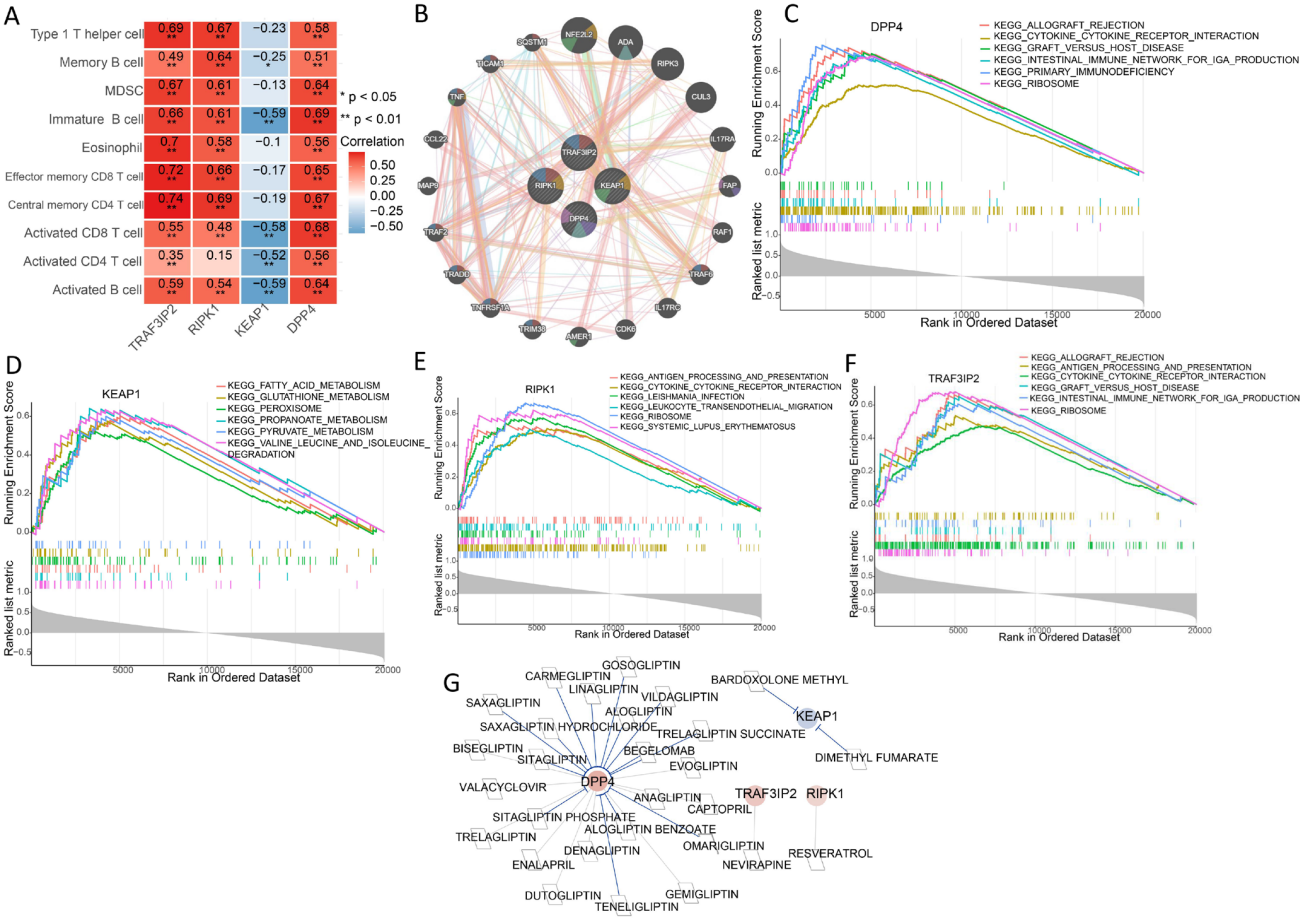
PPI network, correlation between four diagnostic genes and differential immune cells, GSEA, and drug sensitivity analysis of diagnostic genes (A) The correlation between 4 diagnostic genes and differential immune cells. (B) PPI analysis of 4 diagnostic genes and 20 interacting genes. GSEA analysis of 4 diagnostic genes, including DPP4 (C), KEAP1 (D), RIPK1 (E), and TRAF3IP2 (F). (G) Drug sensitivity analysis.

The association between four diagnostic genes and differential immune cells was shown in Figure 7A. TRAF3IP2, RIPK1, and DPP4 were positively correlated with all differentially infiltrated immune cells, while KEAP1 was negatively correlated with memory B cells, immature B cells, activated CD8 T cells, activated CD4 T cells, and activated B cells. In addition, the PPI analysis of four diagnostic biomarkers and 20 interacting genes was conducted; the results showed that these genes were mainly involved in immune‐related pathways (Figure 7B). GSEA analysis showed that DPP4 was positively correlated with 42 KEGG pathways such as cytokine–cytokine receptor interaction and negatively correlated with one KEGG pathway as vasopressin‐regulated water reabsorption (Figure 7C). KEAP1 was enriched in 55 positively correlated KEGG pathways such as fatty acid metabolism and 6 negatively correlated KEGG pathways such as primary immunodeficiency (Figure 7D). RIPK1 was positively correlated with 48 KEGG pathways and negatively correlated with 7 KEGG pathways (Figure 7E). TRAF3IP2 was positively correlated with 57 KEGG pathways and negatively correlated with 7 KEGG pathways (Figure 7F). Besides, total of 28 drug–gene interactions were obtained based on the DGIdb database, and the drug–gene network with four diagnostic genes and 28 small‐molecule drugs was constructed (Figure 7G).

### 3.7 Validation Analysis in Clinical Samples

**FIGURE 8:**
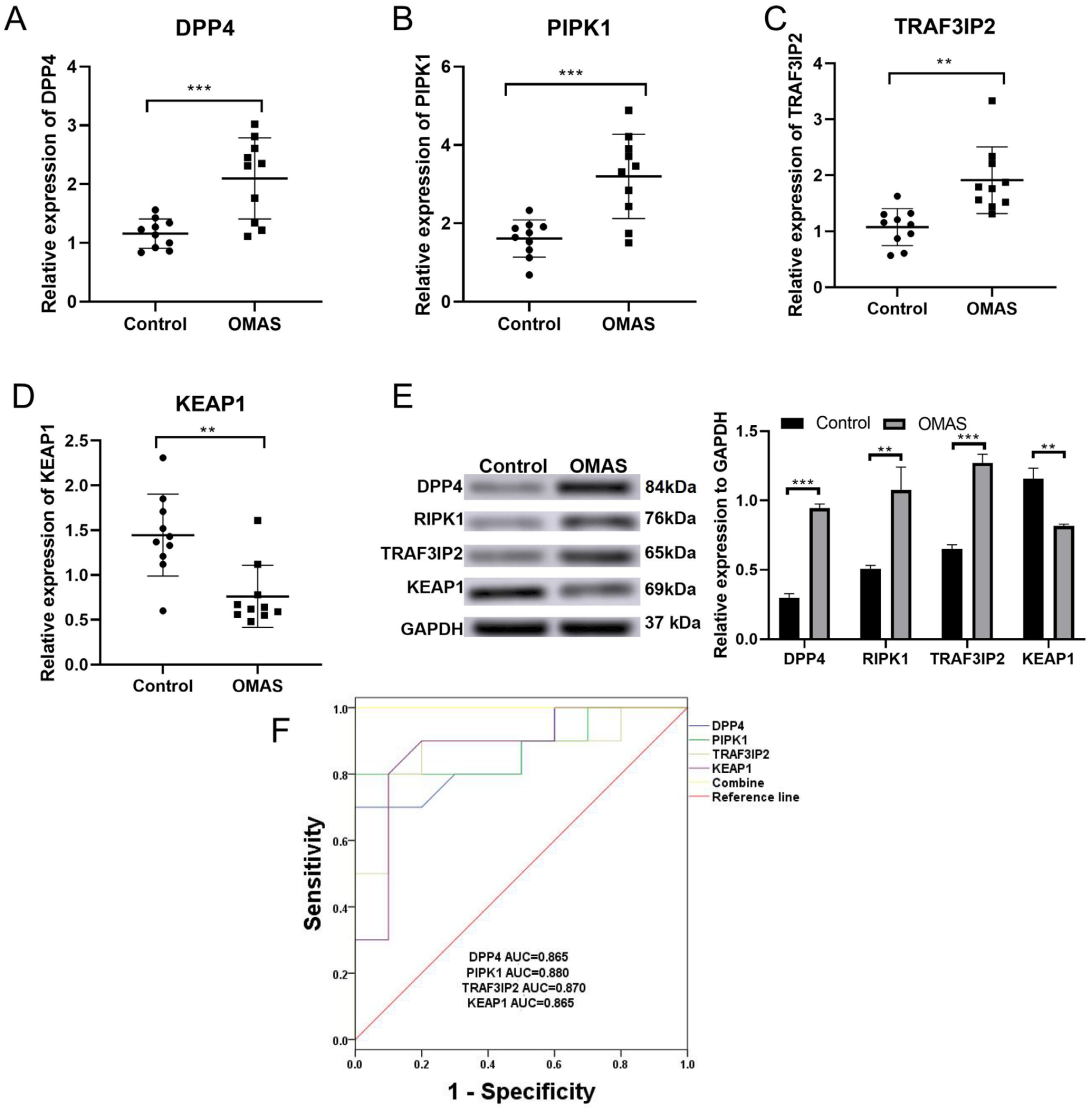
Validation analysis in clinical samples. RT‐qPCR analysis of the mRNA expression of DDP4 (A), PIPK1 (B), RTAF3IP2 (C), and KEAP1 (D). (E) Western blot analysis of the protein expression of biomarkers. (F) ROC curve of the biomarkers in distinguishing control and OMAS patients.

Finally, the differential expression of four diagnostic genes (DPP4, KEAP1, RIPK1, and TRAF3IP2) was verified using qRT‐PCR and western blot. As shown in Figure 8A–E, the assays confirmed that DPP4, RIPK1, and TRAF3IP2 were significantly upregulated at mRNA and protein levels in the OMAS group, while KEAP1 was significantly downregulated when compared to the control group. The ROC curve showed that the four biomarkers exerted high discriminative power for distinguishing OMAS patients from non‐OMAS ones, with the AUCs > 0.8 (Figure 8F). Comparison of the basic information of patients between the two groups revealed no significant difference in age and neuroblastoma location. However, the OMAS group showed a lower male‐to‐female ratio than non‐OMAS patients and a larger number of patients with hepatitis A/meningococcal vaccination injection (Table 2). These indicated that male sex, history of hepatitis A/meningococcal vaccination injection, and the differential expression of biomarkers could be risk factors for OMAS patients in neuroblastoma.

**TABLE 2:** Comparison of patient baseline data.

Feature	Non‐OMAS (*n* = 10)	OMAS (*n* = 10)	*t*/*χ* ^2^	*p*
Age (years)	6.70 ± 2.11	7.20 ± 1.93	−0.553	0.587
Gender (male/female)	7/3	2/8	5.051	0.025
History of hepatitis A and meningococcal vaccination (no/yes)	6/4	1/9	5.495	0.019
Location of neuroblastoma (non‐abdominal/abdominal)	4/6	5/5	0.202	0.653
DPP4 expression	1.16 ± 0.25	2.10 ± 0.69	4.047	< 0.001
PIPK1 expression	1.61 ± 0.47	3.20 ± 1.08	4.267	< 0.001
TRAF3IP2 expression	1.08 ± 0.33	1.91 ± 0.96	3.904	0.001
KEAP1 expression	1.45 ± 0.46	0.76 ± 0.35	3.761	0.001

## 4 Discussion

OMAS is a rare disorder in the nervous system of children, of which most cases were associated with neuroblastoma [24]. Despite some advances, the diagnosis and treatment of OMAS remain challenging. The pathogenesis of OMAS is determined to be paraneoplastic immune‐mediated encephalopathy. Herein, we aimed to explore the immune metabolism diagnostic biomarkers for OMAS in neuroblastoma.

In this study, a series of integrated bioinformatics analyses and machine learning methods were applied to screen the diagnostic genes and assess the diagnostic value for OMAS patients with neuroblastoma. Finally, four pivotal immune‐associated candidate genes were identified, including TRAF3IP2, RIPK1, KEAP1, and DPP4. RIPK1 is a serine/threonine kinase, critical in cell signaling transduction and cell survival [25]. RIPK1 facilitates neuroinflammation and cell death, playing a crucial role in the development of various neurodegenerative disorders [26]. Microglia activation in OMAS correlates with neuronal death [27]. RIPK1 is a critical mediator of microglial dysfunction in neurodegenerative diseases [28] and serves as a potential therapeutic target for treating neurodegenerative diseases [29]. Although there was no direct evidence of the significant role of RIPK1 in OMAS, it is inferred that RIPK1 may be implicated in microglial dysfunction in OMAS. TRAF3 interacting protein 2 (TRAF3IP2) is reported to have participated in the progression of Alzheimer's disease, a neurodegenerative condition [30]. Hu et al. [ 30] have suggested that TRAF3IP2 was highly expressed in AD and exerted promising diagnostic performance for ad, which was consistent with our results. TRAF3IP2‐AS1 was overexpressed in the nervous system of multiple sclerosis, which mediated the autoimmune and inflammatory response [31]. The differential expression of TRAF3IP2‐AS1 was consistent with our results. Given that OMAS is an autoimmune disorder, TRAF3IP2 overexpression may play a key role in the immune and inflammatory response in OMAS. Moreover, KEAP1 acts as an adaptor for the CUL3‐based ubiquitin E3 ligase, which amplifies the ubiquitination of NRF2, leading to its degradation [32]. Tanji et al. [33] discovered that KEAP1 is located within cytoplasmic inclusions in neurons and glial cells in several neurodegenerative diseases. Under stressful conditions, cysteine residues in KEAP1 are modified, leading to a decrease in the degradation of NRF2, which allows it to accumulate and trigger the expression of target genes. This regulatory mechanism is known as the KEAP1‐NRF2 system [34]. Targeting the KEAP1‐NRF2 system was proposed as a promising strategy for treating neurodegenerative diseases. The KEAP1‐NRF2 signaling, as an antioxidant pathway, plays a key role in controlling inflammation of multiple organs. KEAP1 serves as the drug target for inflammatory diseases [35]. Suzuki et al. [36] suggested that KEAP1 knockdown relieved autoimmune inflammation in scurfy mice. Thus, the KEAP1‐NRF2 system may be a promising target for treating OMAS‐associated inflammation. Furthermore, DPP4 inhibitors have demonstrated effectiveness in mitigating neuronal degeneration and improving motor function in multiple preclinical and clinical studies of Parkinson's disease [37]. DPP4, as a moonlighting protein, plays an emerging role in autoimmune disease [38]. Inhibition of DPP4 reduced autoimmune inflammation by the TGF‐β related pathway in autoimmune diabetes [38]. Similarly, considering that all the biomarker genes were closely associated with inflammation and autoimmune diseases, we speculated that biomarkers may play key roles in the pathogenesis of OMAS by mediating the autoimmune response. This study found that compared with non‐OMAS patients, the male‐to‐female ratio in the OMAS group was lower, and the number of patients who received the hepatitis A/meningococcal vaccination injection was larger. A previous study investigated the clinical profile of childhood OMAS and found that among 14 patients, the male‐to‐female ratio was 1:2.3, and only 2 patients received vaccination [39]. Additionally, an ROC curve was drawn to assess the diagnostic value of biomarker genes; the results showed that AUCs of four biomarker genes were all above 0.8, indicating good diagnostic performance for OMAS patients, which was also validated in clinical samples. All these implied the reliability and robustness of our results. Therefore, the four diagnostic genes could be utilized as diagnostic targets for OMAS patients.

The activation of eosinophils and the production of soluble mediators such as IgE antibodies are key pathophysiological features of allergic diseases [40]. Liu et al. [41] uncovered that the proportion of eosinophils in the Alzheimer's disease group was lower than that of the normal group. Besides, granulocytic myeloid‐derived suppressor cells play a vital role in controlling autoimmune disorders within the central nervous system [42]. Knier et al. [43] have found that myeloid‐derived suppressor cells regulate the accumulation of B cells within the central nervous system during autoimmune reactions. In addition, Marcondes et al. [44] have suggested that highly activated CD8 T cells present in the brain are associated with early dysfunction of the central nervous system during simian immunodeficiency virus infection. This study also found that these four diagnostic genes were associated with various immune cells, such as eosinophils, myeloid‐derived suppressor cells, and activated CD8 T cells. Meanwhile, these four diagnostic genes were involved in immune‐related pathways. In summary, infiltrating immune cells play a role in the occurrence and progression of OMAS. Targeting TRAF3IP2, RIPK1, KEAP1, and DPP4 to improve abnormal immune status may be a potential therapeutic approach for treating OMAS in children.

In order to explore the drug chemistry of small molecules related to diagnostic genes, the “DGIdb” database was applied to search for targeted drugs for the four diagnostic genes. The results showed that total of 28 drug–gene interactions were obtained, including diagnostic genes and 28 drug small molecules. The targeted drugs for the four diagnostic genes contained bardoxolone methyl, alogliptin, and teneligliptin. Bardoxolone methyl is an NRF2 activator, which regulates multiple oxidative and inflammatory diseases [45]. Takagi et al. [46] have found that earlier NRF2 activation may protect neurons, possibly through its effects on astrocytes. Thus, bardoxolone methyl may have a protective effect on neurons. In addition, Rahman et al. [47] showed that in an animal model of Alzheimer's disease induced by amyloid‐beta fibrils, alogliptin was found to reverse insulin resistance in the hippocampus. Safar et al. [48] uncovered that alogliptin has a promising neuroprotective effect in Parkinson's disease. Besides, Guo et al. [49] revealed that canagliflozin combined with teneligliptin on β‐cell volume density and diabetic polyneuropathy in spontaneously type 2 diabetic Goto‐Kakizaki rats shows beneficial effects. Therefore, bardoxolone methyl, alogliptin, and teneligliptin might be used for OMAS treatment in children by targeting TRAF3IP2, RIPK1, KEAP1, and DPP4.

The study has several limitations. First, this study only relied on the GSE189367 dataset and had a small number of clinical samples, which limited the general applicability of the research results. Second, the screened immune cells and small‐molecule drugs should be further tested through experimental analyses. Third, the diagnostic value of the nomogram and diagnostic model should be validated in other datasets. Therefore, in the subsequent research, we plan to incorporate more clinical samples and conduct further in vitro and in vivo functional experiments, such as cell‐based analysis and animal model studies, to investigate how these genes affect the pathological progression of OMAS and the potential synergistic or regulatory relationships among them.

This study discovered four immune metabolism‐associated diagnostic genes (TRAF3IP2, RIPK1, KEAP1, and DPP4) and developed a reliable diagnostic model and a nomogram for predicting OMAS in children. These discoveries offer valuable insights into the diagnosis and targeted therapies for OMAS in children.

## Ethics Statement

All human studies have been reviewed by the ethics committees of Weifang People's Hospital (KYLL20220622‐2) and have all met the ethical standards stipulated in the Helsinki Declaration (Brazilian version, 2013, revised edition).

## Consent

Informed consent was obtained from all individual participants included in the study.

## Conflicts of Interest

The authors declare no conflicts of interest.

## Supporting information


**Appendix S1:** A list of 162 differential immune metabolic genes.

## Data Availability

The data that support the findings of this study are available on request from the corresponding author. The data are not publicly available due to privacy or ethical restrictions.
