# Supplementary material for: Identification and Verification of Immune Metabolism–Related Biomarkers and Immune Infiltration Landscape for Pediatric Opsoclonus Myoclonus Ataxia Syndrome in Neuroblastoma
Source: CNS Neurosci Ther. 2025 Nov 4;31(11):e70610. doi: 10.1111/cns.70610 (PMC12586342; doi:10.1111/cns.70610)
Supplement: Supplementary file 1 — Appendix S1: A list of 162 differential immune metabolic genes. [file CNS-31-e70610-s001.pdf]

Full unedited gel/blot for Figure 7E

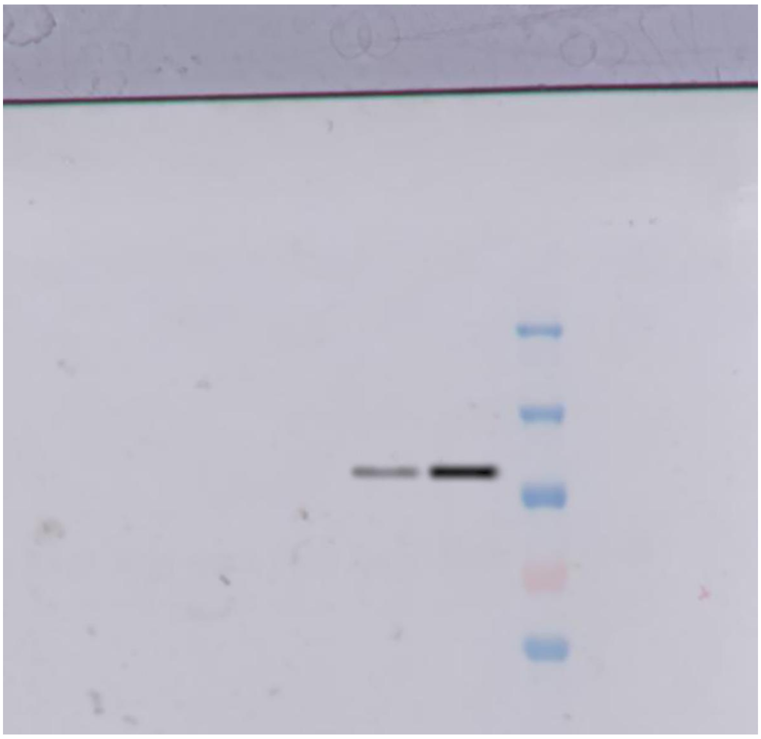

raw blot for DPP4-84 kDa

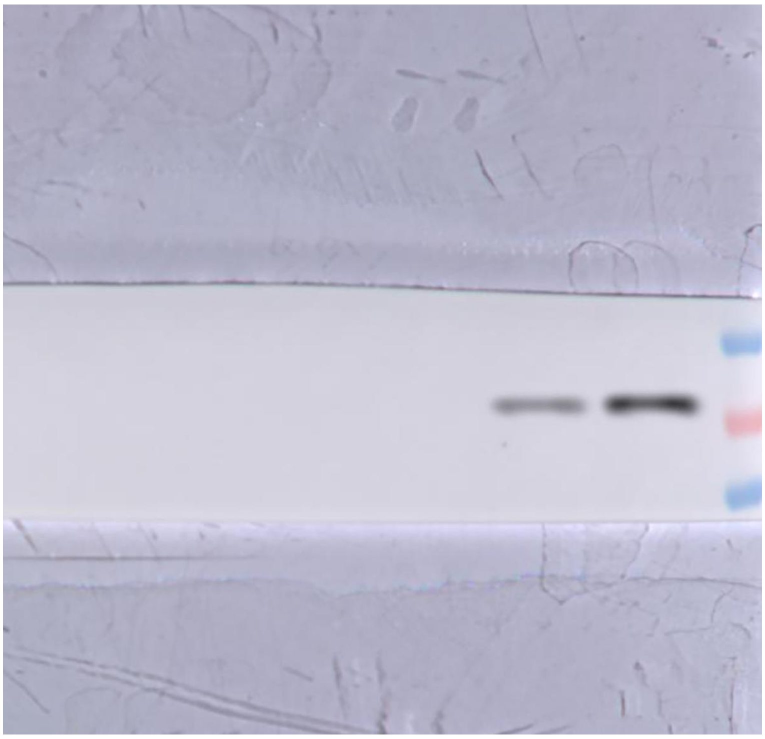

raw blot for RIPK1-76 kDa

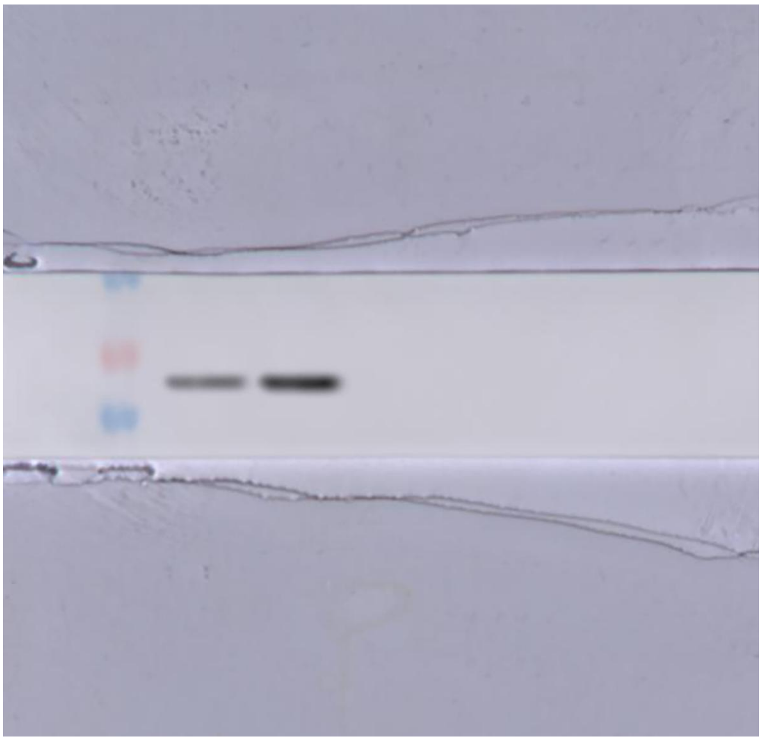

raw blot for TRAF3IP2-65 kDa

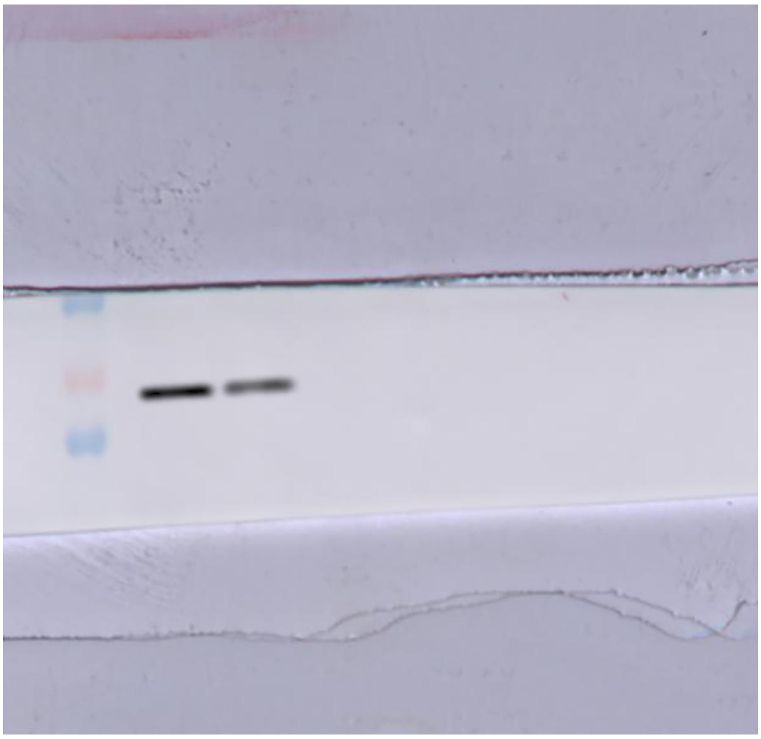

raw blot for KEAP1-69 kDa

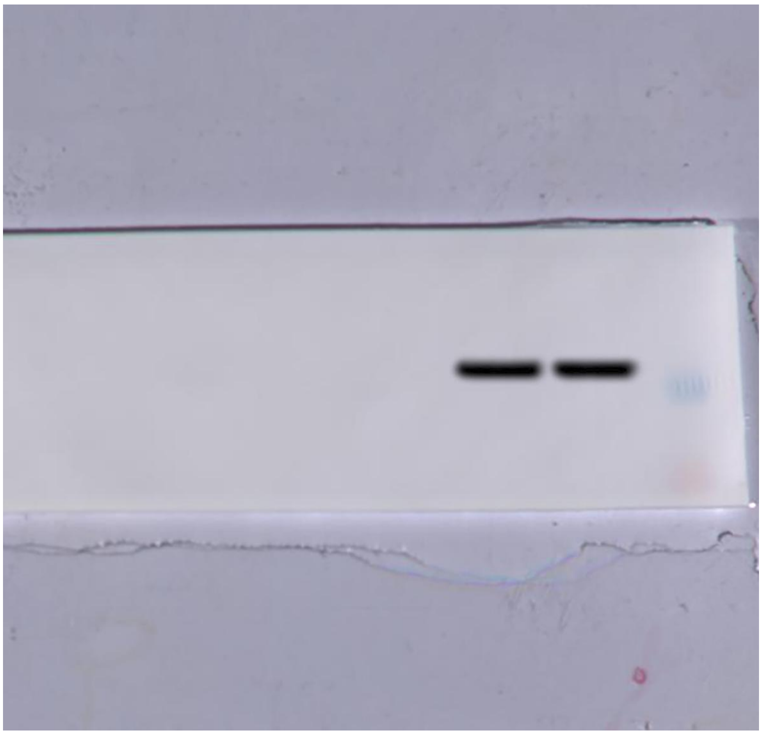

raw blot for GAPDH-37 kDa
